# Bamboo Leaf Sign as a Sensitive Magnetic Resonance Imaging Finding in Spinal Subependymoma: Case Report and Literature Review

**DOI:** 10.1155/2016/9108641

**Published:** 2016-12-15

**Authors:** Hiroyuki Toi, Yukari Ogawa, Keita Kinoshita, Satoshi Hirai, Hiroki Takai, Keijiro Hara, Nobuhisa Matsushita, Shunji Matsubara, Masaaki Uno

**Affiliations:** Department of Neurosurgery, Kawasaki Medical School, Kurashiki, Okayama, Japan

## Abstract

*Background and Importance*. Subependymoma occurs very rarely in the spinal cord. We report another case of spinal subependymoma along with a review of the literature and discussion of a radiological finding that is useful for preoperative diagnosis of this tumor.* Clinical Presentation*. A 51-year-old man presented with a 2-year history of progressive muscle weakness in the right lower extremity. Sagittal magnetic resonance imaging (MRI) showed spinal cord expansion at the Th7–12 vertebral level. Surgical resection was performed and the tumor was found to involve predominantly subpial growth. Histological diagnosis was subependymoma, classified as Grade I according to criteria of World Health Organization. We made an important discovery of what seems to be a characteristic appearance for spinal subependymoma on sagittal MRI. Swelling of the spinal cord is extremely steep, providing unusually large fusiform dilatation resembling a bamboo leaf. We have termed this characteristic MRI appearance as the “bamboo leaf sign.” This characteristic was apparent in 76.2% of cases of spinal subependymoma for which MRI findings were reported.* Conclusion*. The bamboo leaf sign on spinal MRI is useful for differentiating between subependymoma and other intramedullary tumors. Neurosurgeons encountering the bamboo leaf sign on spinal MRI should consider the possibility of subependymoma.

## 1. Background and Importance

Subependymoma, first described by Scheinker [1] in 1945, is a rare central nervous system tumor, accounting for 0.7% of all intracranial tumors [2]. Subependymoma is a relatively slow-growing benign tumor corresponding histologically to World Health Organization (WHO) Grade I [3]. About 50% of these tumors are clinically silent and only found incidentally at autopsy [3, 4], with the remaining 50% presenting symptomatically during life. This pathology is most frequently identified in the fourth ventricle (50–60%), followed by the lateral ventricles (30–40%) [3–6], and occurs very rarely in the spinal cord. To the best of our knowledge, only 54 cases of spinal subependymomas have been reported in the literature [2, 7–30]. We report here another case of spinal subependymoma with a review of the literature and the new radiological findings, which may be useful for a preoperative diagnosis of this tumor.

## 2. Clinical Presentation

A 51-year-old man presented with a 2-year history of progressive muscle weakness in the right lower extremity and dysesthesia in both lower extremities. Neurological examination revealed severe loss of all sensory modalities below the L1 dermatomes. Motor examination revealed monoparesis of the right lower limbs, and muscle power assessment showed Grade 1/5 in tibialis anterior, extensor hallucis longus, gastrocnemius, and flexor hallucis longus in Manual Muscle Testing. Deep tendon reflexes in both lower limbs were hyperactive. He presented with slight bladder and rectal disturbance of constipation and inability to urinate.

### 2.1. Neuroimaging Findings

**Figure 1:**
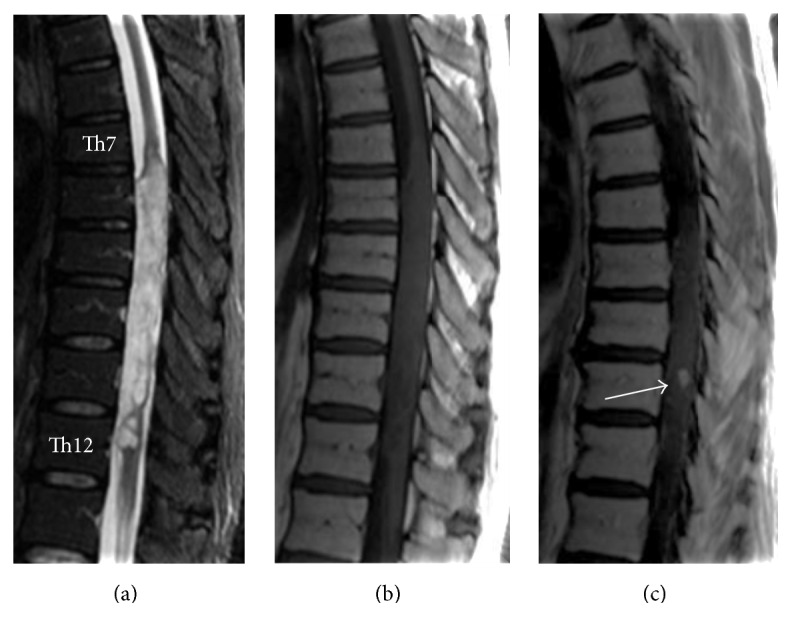
(a) Sagittal T2W1 showing cord expansion and hyperintense signal extending from the Th7 level to Th12 level surrounding both anterior and posterior aspects of cord. (b) Sagittal T1W1 showing an isointense mass with no clear-cut demarcation between cord and tumor. (c) Gadolinium contrast-enhanced MRI reveals slight enhancement. This is only a part of the tumor.

**Figure 2:**
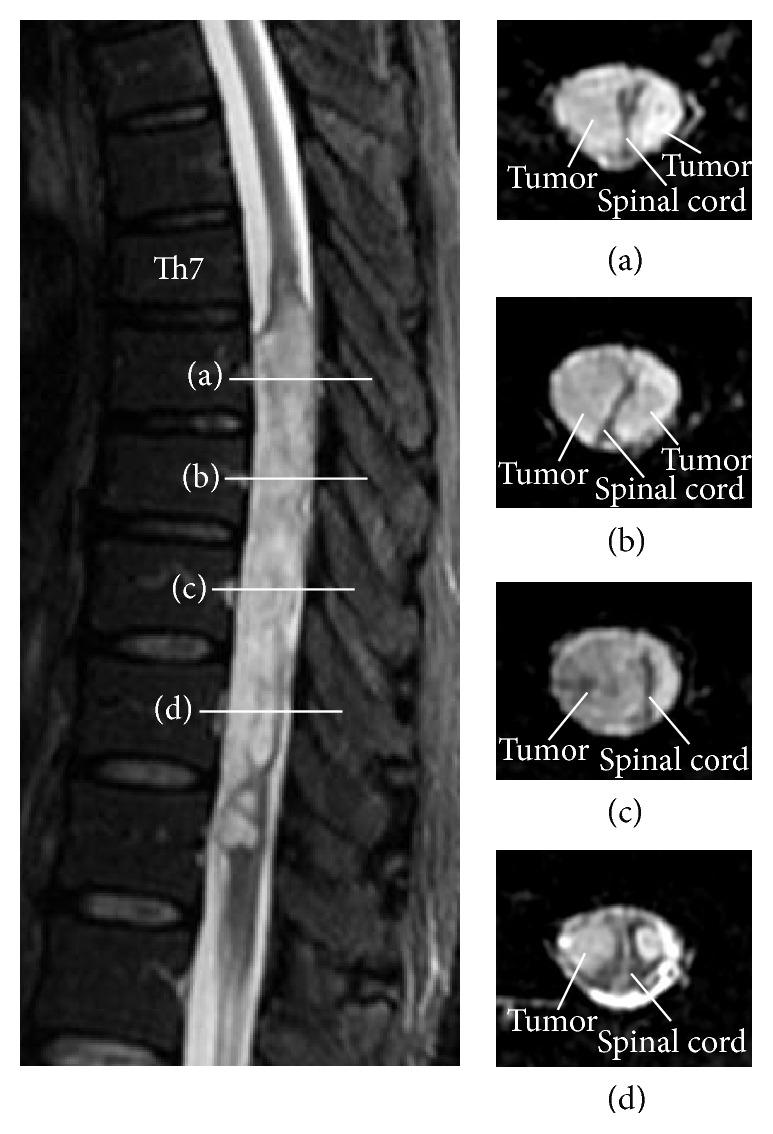
Axial T2WI at the Th8–11 level, showing an eccentric intramedullary tumor located bilaterally. Bars indicate levels of axial views for Th8 (a), Th9 (b), Th10 (c), and Th11 (d).

Sagittal images from T2-weighted imaging (T2WI) (Figure 1(a)) showed cord expansion and a hyperintense signal extending from the Th7 to Th12 vertebral level, surrounding both anterior and posterior aspects of the cord. Sagittal images from T1-weighted imaging (T1WI) (Figure 1(b)) revealed an isointense mass lesion with no clear-cut demarcation of the interface between the spinal cord and tumor. A small enhanced portion was seen with gadolinium administration (Figure 1(c)). Axial T2WI showed extension of the tumor along bilateral surfaces of the spinal cord with strong compression (Figure 2). The spinal cord was severely deformed from compression by the tumor. At the marginal portion of the tumor, cord expansion was extremely steep. Imaging findings were considered to favor an intramedullary tumor, and differential diagnoses of ependymoma and astrocytoma were considered.

### 2.2. Operative Intervention and Findings

A two-stage operation for resection of the tumor was performed. The first surgery included Th7–Th12 laminectomy and removal of half of the tumor. Two weeks postoperatively, a second surgery was performed to remove the residual tumor. The tumor was found to be predominantly subpial, showing an eccentric position with an indistinct plane between the tumor and cord. The tumor was soft, greyish, and avascular and was lying bilaterally alongside the spinal cord. Radical tumor decompression was performed and subtotal resection was achieved.

### 2.3. Histopathological Findings

The tumor tissue was fixed in 10% neutral-buffered formalin, routinely processed, and embedded in paraffin. Sections of 5 *μ*m thickness were cut, stained with hematoxylin and eosin, and examined. The tumor comprised cells with fine processes and round-to-ovoid nuclei, arranged in clusters within microcystic backgrounds. No ependymal rosette formation was seen. Morphological features of malignancy, such as cellular anaplasia, necrosis, and increased mitosis, were absent. MIB-I labeling index was very low (0.1%). Based on these features, a histological diagnosis of subependymoma (WHO Grade I) was made.

### 2.4. Postoperative Course

Postoperatively, the patient developed paraparesis and severe bladder and rectal disturbance. Muscle power assessment of the lower limbs showed Grade 2/5. No delayed complications or tumor recurrence occurred during the follow-up period of 24 months.

## 3. Discussion

Spinal subependymoma is much less frequent than intracranial subependymoma [3–7, 13, 31]. Since the initial description of spinal cord subependymoma by Boykin et al., 54 cases have been reported in the literature [1–48]. Most reports have involved single cases; no large series have been described.

Spinal subependymoma is a slow-growing and noninvasive benign tumor, accounting for 1~2% of all spinal ependymal tumors [32]. This pathology corresponds histologically to WHO Grade I [33]. The age of patients at diagnosis has ranged from 6 to 73 years, with a mean of 43.6 years. A male preponderance has been noted (males : females = 3 : 2) [1–48]. The majority of these tumors have shown an intramedullary location, within the cervical (26 cases), thoracic (22 cases), or lumbar region (2 cases). One patient displayed holocord subependymoma. In 3 cases, location of the tumor was not described [9].

The majority of spinal subependymomas originate from intra-axial tissue. A distinctive feature noted at surgery is the eccentric subpial location of these subependymomas, in contrast with the central location of ependymomas and astrocytomas. A number of reports have described subependymoma exhibiting subpial growth.

**Figure 3:**
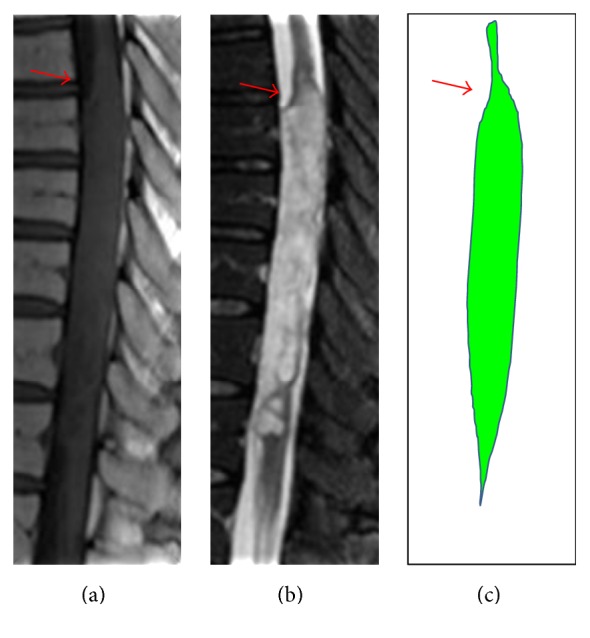
Bamboo leaf sign. The figure shows MRI of spinal subependymoma from our case. (a) T1WI. (b) T2WI. Sagittal MRI shows steep swelling of the spinal cord (arrows) and unusually large fusiform dilatation, resembling the shape of a bamboo leaf (c).

**Figure 4:**
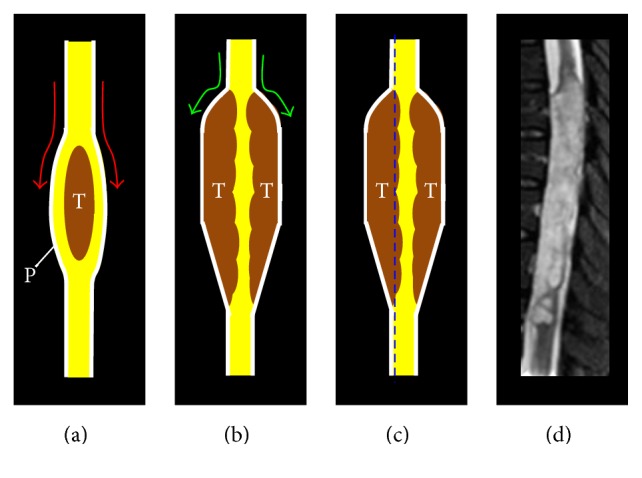
Schematic representation of MRI findings for spinal subependymoma demonstrating the bamboo leaf sign. (a) Other intramedullary tumors like ependymoma and astrocytoma show gradual dilatation of the spinal cord. (b) Spinal subependymoma shows steep swelling of the spinal cord due to subpial growth. (c) Blue dashed line shows sagittal section for MRI of the spinal subependymoma. (d) Sagittal T2WI of subependymoma showing bamboo leaf sign. T, tumor; P, pia mater.

This tumor is difficult to differentiate from spinal astrocytoma or ependymoma on the basis of clinical and radiological findings. No pathognomonic features distinctive for subependymoma have previously been reported on MRI. We report for the first time a specific feature of spinal subependymoma on MRI which may help distinguish this tumor from other intramedullary tumors, such as astrocytoma or ependymoma. We made an important discovery of a characteristic appearance common to spinal subependymomas on sagittal MRI. Swelling of the spinal cord is extremely steep, taking the form of an unusually large fusiform dilatation resembling a bamboo leaf on sagittal MRI (Figure 3). We have therefore termed this characteristic appearance as the “bamboo leaf sign” (Figure 4). MRI findings have been described for 21 cases reported since 1995 (including the present case), with 16 cases (76.2%) displaying the bamboo leaf sign (Table 1). This sign is the product of the steep swelling of the spinal cord resulting from subpial growth of the tumor (Figure 4).

Spinal astrocytoma and ependymoma do not show the bamboo leaf sign due to their central location in the spinal cord. MRI instead reveals gradual fusiform dilatation of the spinal cord with these tumors. The bamboo leaf sign thus appears useful for differentiating between subependymoma and other intramedullary tumors, although careful differentiation is needed for subpial growing tumors such as hemangioblastomas and intramedullary infection.

## 4. Conclusion

Due to the rarity of spinal subependymoma, this pathology may be omitted from preoperative differential diagnosis; however, awareness of the existence of these tumors within the spinal cord is necessary. It is important for neurosurgeons and pathologists to recognize and diagnose this entity and to differentiate it from both astrocytoma and ependymoma. The importance of correct diagnosis lies in the fact that these tumors are completely benign, and total resection will achieve complete cures without the need for adjuvant therapy. When total resection is impossible due to a lack of demarcation from the cord or tumor infiltration into the surrounding cord, aggressive treatment should be avoided. Even if the tumor remains, long-term prognosis is considered good because of the benign behavior of this pathology. For neurosurgeons encountering the bamboo leaf sign on spinal MRI, the possibility of subependymoma should be kept in mind.

## Figures and Tables

**Table 1:** Summary of cases of spinal subependymomas reported since 1995.

Author, year	Number of reported cases	MRI available cases	Tumor location (MRI available cases)	Bamboo leaf sign
Hoeffel et al., 1995	1	1	C1–5	+
Tacconi et al., 1996	1	1	C6-7	+
Jallo et al., 1996	6	3	C6-Th1/C4-Th1/C7-Th1	+/+/−
Dario et al., 2001	1	1	Th9-L1	+
Matsumoto and Nakagaki, 2002	1	1	Th2–7	+
Sarkar et al., 2003	1	1	C3–7	−
Shimada et al., 2003	2	1	Th5–8	+
Kremer et al., 2004	1	1	Th11-L2	+
Fukuzumi et al., 2006	1	1	Th3–7	+
Yadav et al., 2008	1	1	Th5–9	+
Jang et al., 2009	1	1	Th11-12	−
Orakcioglu et al., 2009	2	2	Th10-l2/Th11-L1	+/+
Zenmyo et al., 2010	1	1	C1-2	−
Jabri et al., 2010	1	1	Th10-L1	+
Yamamoto, 2010	4	1	C6-Th5	+
Krishnan et al., 2012	1	1	C3-Th4	+
Iwasaki et al., 2013	1	1	Th11-12	−
Our case, 2013	1	1	Th7–12	+

Total	28	21	C: 8 cases/Th: 13 cases	16 (76.2%)

## References

[1] Scheinker I. M. (1945). Subependymoma: a newly recognized tumor of subependymal derivation. *Journal of Neurosurgery*.

[2] Tacconi L., Johnston F. G., Thomas D. G. T. (1996). Subependymoma of the cervical cord. *Clinical Neurology and Neurosurgery*.

[3] Wiestler O. D., Schiffer D., Kleihues P., Cavenee W. K. (2000). Subependymoma. *Pathology & Genetics: Tumours of the Nervous System*.

[4] Scheithauer B. W. (1978). Symptomatic subependymoma. Report of 21 cases with review of the literature. *Journal of Neurosurgery*.

[5] Jooma R., Torrens M. J., Bradshaw J., Brownell B. (1985). Subependymomas of the fourth ventricle. Surgical treatment in 12 cases. *Journal of Neurosurgery*.

[6] Ironside J. W., Moss T. H., Louis D. N., Lowe J. S., Weller R. O. (2002). Ependymal and choroid plexus tumours. *Diagnostic Pathology of Nervous System Tumours*.

[7] Boykin F. C., Cowen D., Iannucci C. A., Wolf A. (1954). Subependymal glomerate astrocytomas. *Journal of Neuropathology and Experimental Neurology*.

[8] Slowik F., Pásztor E., Szöllösi B. (1979). Subependymal gliomas. *Neurosurgical Review*.

[9] Pluchino F., Lodrini S., Lasio G., Allegranza A. (1984). Complete removal of holocord subependymoma. Case report. *Acta Neurochirurgica*.

[10] Salcman M., Mayer R. (1984). Intramedullary subependymoma of the cervical spinal cord: case report. *Neurosurgery*.

[11] Cervos-Navarro J., Artigas J., Perez-Canto A. (1986). Clinical and immunohistological findings in subependymomas of the spinal cord. *Verhandlungen der Deutschen Gesellschaft fur Pathologie*.

[12] Lee K. S., Angelo J. N., McWhorter J. M., Davis C. H. (1987). Symptomatic subependymoma of the cervical spinal cord. Report of two cases. *Journal of Neurosurgery*.

[13] Matsumura A., Hori A., Spoerri O. (1988). Spinal subependymoma presenting as an extramedullary tumor: case report. *Neurosurgery*.

[14] Bardella L., Artico M., Nucci F. (1988). Intramedullary subependymoma of the cervical spinal cord. *Surgical Neurology*.

[15] Nagashima M., Isu T., Iwasaki Y. (1988). Intramedullary subependymoma of the cervical spinal cord. Case report. *Neurologia Medico-Chirurgica*.

[16] Vaquero J., Martinez R., Vegazo I., Ponton P. (1989). Subependymoma of the cervical spinal cord. *Neurosurgery*.

[17] Guha A., Resch L., Tator C. H. (1989). Subependymoma of the thoracolumbar cord. Case report. *Journal of Neurosurgery*.

[18] Lach B., Russell N., Benoit B. (1990). Atypical subependymoma of the spinal cord: ultrastructural and immunohistochemical studies. *Neurosurgery*.

[19] Bergman T. A., Haines S. J. (1991). Subependymoma of the cervical spinal cord. A case report of long-term survival. *Minnesota Medicine*.

[20] Tomlinson F. H., Scheithauer B. W., Kelly P. J., Forbes G. S. (1991). Subependymoma with rhabdomyosarcomatous differentiation: report of a case and literature review. *Neurosurgery*.

[21] Nakasu S., Nakasu Y., Saito A., Handa J. (1992). Intramedullary subependymoma with neurofibromatosis. Report of two cases. *Neurologia Medico-Chirurgica*.

[22] Pagni C. A., Canavero S., Giordana M. T., Mascalchi M., Arnetoli G. (1992). Spinal intramedullary subependymomas: case report and review of the literature. *Neurosurgery*.

[23] Salvati M., Raco A., Artico M., Artizzu S., Ciappetta P. (1992). Subependymoma of the spinal cord. Case report and review of the literature. *Neurosurgical Review*.

[24] Casey A. T. H., Marsh H., Wilkins P. (1993). Subependymoma of the thoracic cord: potential pitfalls in diagnosis. *British Journal of Neurosurgery*.

[25] Polivka M., Lot G., Woimant F., Lavergne A., Chedru F., Mikol J. (1994). Seven cases of subependymoma. Anatomoclinical study and review of the literature. *Archives d'Anatomie et de Cytologie Pathologiques*.

[26] Roeder M. B., Jinkins J. R., Bazan C. (1994). Subependymoma of filum terminale: MR appearance. *Journal of Computer Assisted Tomography*.

[27] Hoeffel C., Boukobza M., Polivka M. (1995). MR manifestations of subependymomas. *American Journal of Neuroradiology*.

[28] Jallo G. I., Zagzag D., Epstein F. (1996). Intramedullary subependymoma of the spinal cord. *Neurosurgery*.

[29] Bret P., Bougeard R., Saint-Pierre G., Guyotat J., Ricci A.-C., Confavreux C. (1997). Intramedullary subependymoma of the cervical spinal cord. Review of the literature a propos of a case. *Neurochirurgie*.

[30] Dario A., Fachinetti P., Cerati M., Dorizzi A. (2001). Subependymoma of the spinal cord: case report and review of the literature. *Journal of Clinical Neuroscience*.

[31] Schiffer D., Chiò A., Giordana M. T. (1991). Histologic prognostic factors in ependymoma. *Child's Nervous System*.

[32] Boström A., von Lehe M., Hartmann W. (2011). Surgery for spinal cord ependymomas: outcome and prognostic factors. *Neurosurgery*.

[33] Louis D. N., Ohgaki H., Wiestler O. D., Cavenee W. K. (2007). *WHO Classification of Tumours of the Central Nervous System*.

[34] Artico M., Bardella L., Ciappetta P., Raco A. (1989). Surgical treatment of subependymomas of the central nervous system. Report of 8 cases and review of the literature. *Acta Neurochirurgica*.

[35] Fukuzumi Y., Tani S., Isoshima A., Nagashima H., Abe T., Fujigasaki J. (2006). Spinal cord subependymoma—a case report and review of the literature. *Spinal Surgery*.

[36] Herrmann H.-D., Neuss M., Winkler D. (1988). Intramedullary spinal cord tumors resected with CO2 laser microsurgical technique: recent experience in fifteen patients. *Neurosurgery*.

[37] Iwasaki M., Hida K., Aoyama T., Houkin K. (2013). Thoracolumbar intramedullary subependymoma with multiple cystic formation: a case report and review. *European Spine Journal*.

[38] Jabri H. E., Dababo M. A., Alkhani A. M. (2010). Subependymoma of the spine. *Neurosciences*.

[39] Jang W.-Y., Lee J.-K., Lee J.-H. (2009). Intramedullary subependymoma of the thoracic spinal cord. *Journal of Clinical Neuroscience*.

[40] Kremer P., Zoubaa S., Schramm P. (2004). Intramedullary subependymoma of the lower spinal cord. *British Journal of Neurosurgery*.

[41] Krishnan S. S., Panigrahi M., Pendyala S., Rao S. I., Varma D. R. (2012). Cervical Subependymoma: a rare case report with possible histogenesis. *Journal of Neurosciences in Rural Practice*.

[42] Matsumoto K., Nakagaki H. (2002). Intramedullary subependymoma occupying the right half of the thoracic spinal cord—case report. *Neurologia Medico-Chirurgica*.

[43] Orakcioglu B., Schramm P., Kohlhof P., Aschoff A., Unterberg A., Halatsch M.-E. (2009). Characteristics of thoracolumbar intramedullary subependymomas: clinical article. *Journal of Neurosurgery: Spine*.

[44] Sarkar C., Mukhopadhyay S., Ralte A. M. (2003). Intramedullary subependymoma of the spinal cord: a case report and review of literature. *Clinical Neurology and Neurosurgery*.

[45] Shimada S., Ishizawa K., Horiguchi H., Shimada T., Hirose T. (2003). Subependymoma of the spinal cord and review of the literature. *Pathology International*.

[46] Yadav R. K., Agarwal S., Saini J., Sharma N. K. (2008). Imaging appearance of subependymoma: a rare tumor of the cord. *Indian Journal of Cancer*.

[47] Yamamoto S. (2010). Characteristics of intramedullary subependymoma. *Spinal Surgery*.

[48] Zenmyo M., Ishido Y., Terahara M. (2010). Intramedullary subependymoma of the cervical spinal cord: a case report with immunohistochemical study. *International Journal of Neuroscience*.

